# Evaluation of hematological changes and immune response biomarkers as a prognostic factor in critical patients with COVID-19

**DOI:** 10.1371/journal.pone.0297490

**Published:** 2024-02-29

**Authors:** Liliane Rosa Alves Manaças, Robson Luís Oliveira de Amorim, Alian Aguila, Paloam Cardoso Novo, Rebeka Caribé Badin

**Affiliations:** 1 Department of Pharmacology, Brazilian National Cancer Institute (INCA), Hospital II, Rio de Janeiro, Rio de Janeiro, Brazil; 2 Department of Neurosurgery, Getúlio Vargas University Hospital, Manaus, Amazonas, Brazil; 3 Department of Cardiology, Memorial Hospital System, Florida, United States of America; 4 Federal University of Amazonas (UFAM), Manaus, AM, Brazil. Post-graduate Program in Basic and Applied Immunology, Institute of Biological Sciences.; Tanta University Faculty of Medicine, EGYPT

## Abstract

COVID-19 disease has been a challenge for health systems worldwide due to its high transmissibility, morbidity, and mortality. Severe COVID-19 is associated with an imbalance in the immune response, resulting in a cytokine storm and a hyperinflammation state. While hematological parameters correlate with prognosis in COVID patients, their predictive value has not been evaluated specifically among those severely ill. Therefore, we aim to evaluate the role of hematological and immune response biomarkers as a prognostic factor in critically ill patients with COVID-19 admitted to the intensive care unit. From May 2020 to July 2021, a retrospective cohort study was conducted in a reference hospital in Manaus, which belongs to the Brazilian public health system. This study was carried out as single-center research. Clinical and laboratory parameters were analyzed to evaluate the association with mortality. We also evaluated the role of neutrophil-to-lymphocyte ratio (NLR), lymphocyte-to-monocyte ratio (LMR), platelet-to-lymphocyte ratio (PLR), and C-reactive protein-to-lymphocyte ratio (CLR). We gathered information from medical records, as well as from prescriptions and forms authorizing the use of antimicrobial medications. During the study period, 177 patients were included, with a mean age of 62.58 ± 14.39 years. The overall mortality rate was 61.6%. Age, mechanical ventilation (MV) requirement, leukocytosis, neutrophilia, high c-reactive protein level, NLR, and CLR showed a statistically significant association with mortality in the univariate analysis. In the multivariate logistic regression analysis, only MV (OR 35.687, 95% CI: 11.084–114.898, p< 0.001) and NLR (OR 1.026, 95% CI: 1.003–1.050, p = 0.028) remained statistically associated with the outcome of death (AUC = 0.8096). While the need for mechanical ventilation is a parameter observed throughout the hospital stay, the initial NLR can be a primary risk stratification tool to establish priorities and timely clinical intervention in patients with severe COVID-19 admitted to the ICU.

## Introduction

COVID-19 represented a significant challenge for health systems worldwide due to its high transmissibility. Clinical symptoms show wide heterogeneity, ranging from asymptomatic infection to aggressive life-threatening complications [1]. As the disease progressed, new variants with increased transmissibility and severity emerged, each wave was represented by a new "Variant of Concern (VOC)" [2]. This disease is multisystemic and highly complex since the severity depends on immunological and genetic factors, the tendency to a prothrombotic state, viral load, and comorbidities [3]. Severe cases of COVID-19 are a result of tissue-directed immunopathology, which causes rapid clinical deterioration and multiple organ dysfunction syndrome (MODS). This is due to a hyperinflammatory response rather than the virus itself being the direct cause [4, 5].

Severe COVID-19 is characterized by a cytokine storm, i.e., uncontrolled systemic hyperinflammation with excessive amounts of pro-inflammatory cytokines (IL-1, IL-6, and TNF-α), which can lead to the failure of multiple organs until death. However, the pathogenesis of this condition has not yet been fully elucidated [6, 7].

Innate immunity is the first line of organism defense against intracellular pathogens. Viral infection promotes the expression of pathogen-associated molecular patterns (PAMPs) that activate immune cells and trigger inflammatory responses.

COVID-19, like other cytopathic viruses, also induces the release of diverse endogenous molecules that acts as a danger-associated molecular pattern (DAMPs). PAMPs and DAMPs derived from the host cellular injury are recognized by pattern recognition receptors (PRRs), such as Toll-like receptors (TLRs), on alveolar macrophages and endothelial cells, promoting the release of pro-inflammatory cytokines and interferon [7]. When inflammation occurs, or the immune system is activated, there are changes in the blood composition. One can assess these changes by conducting different tests, including cytokine dosages, immunoassays, and flow cytometry. However, the routine peripheral blood cell count is a simple, rapid, and cheap test reflecting immune cell subset alterations. The increase in neutrophils and monocyte count represents the inflammatory process and innate immunity. On the other hand, lymphocytes are components of the adaptive immune response [3, 8–10].

Some studies reported that hematological parameters such as white blood cell count (WBC), lymphocyte count, neutrophil count, eosinophil count, platelet count, hemoglobin, neutrophil-lymphocyte ratio (NLR), C-reactive protein (CRP), platelet-lymphocyte ratio (PLR), Lymphocyte-to-monocyte ratio (LMR) and C-reactive protein-to-lymphocyte ratio (CLR) are helpful in stratifying the severity of patients with COVID-19 [11–14]. However, their predictive value has not been explicitly evaluated among those severely ill.

It is imperative to identify rapid, low-cost, and easily accessible biomarkers to recognize high-risk patients as it enables better allocation of resources and early clinical intervention to prevent the mortality related to COVID-19. Therefore, this work aimed to evaluate the role of the hematological parameters, including NLR, LMR, PLR, and CLR in the prognosis of patients with COVID-19 admitted to a Brazilian intensive care unit.

## Materials and methods

### Design study and population

We conducted a retrospective cohort study by analyzing the electronic medical records of patients hospitalized with COVID-19 from May 2020 to July 2021. The extended period of the study intended to include the two exponential waves of COVID-19 cases in the city of Manaus. The cohort was followed up from admission for a maximum period of 180 days, considering the outcomes of discharge or death, whichever occurred first. Data were collected from August to October 2022. The research was carried out at Getúlio Vargas University Hospital ‐ AM’s Intensive Care Unit (ICU), part of the Unified Health System and overseen by the Brazilian Hospital Services Company. The ICU capacity is ten adult beds–TYPE II but was later qualified for 30 adult ICU II beds–severe acute respiratory syndrome (SARS), COVID-19, according to the National Register of Health Establishments (CNES). The Federal University of Amazonas Research Ethics Committee approved the research under protocol number CAAE n˚ 57750422.4.0000.5020. Throughout the study, patients were identified using a growing sequence of alphanumeric codes drawn up by the researchers to ensure the anonymity of the research participants.

### Source data and outcomes

Data collection included medical records, prescriptions, and the electronic system AGHU (Application for Management of University Hospitals). For each patient, we gathered the following information: For each patient, we gathered the following information: demographic (age, and sex), clinical (comorbidities, length of stay, needs of mechanical ventilation, and use of antimicrobials), and laboratory (peripheral blood cell count, and C-reactive protein) data. The clinical outcome of the patients in this research was survivor and non-survivor. The length of stay was set from the date of admission to hospital discharge, transfer to other hospitals or death. The associated hematological parameters are described in Table 1.

**Table 1:** Description of hematological parameters and their formulas.

Parameter	Acronym	Formula
Neutrophil–to–lymphocyte ratio	NLR	(Absolute neutrophil count) / (absolute lymphocyte count
Lymphocyte-to-monocyte ratio	LMR	(Absolute lymphocyte count) / (absolute monocyte count)
Platelet-to-lymphocyte ratio	PLR	(Platelet count) / (absolute lymphocyte count)
C-reactive protein-to-lymphocyte ratio	CLR	(C-reactive protein count) / (absolute lymphocyte count)

### Statistical analysis

We calculated the mean and standard deviation for the quantitative variables, and the Kolmogorov-Smirnov Normality test was applied. Student’s t-test (Normal Distribution) and Mann-Whitney (Non-Normal Distribution) were used to compare two groups (survivors and non-survivors). Categorical variables were shown in percentages or absolute values, and the Chi-Square Test and Fisher’s Exact Test were used to verify the association between categorical variables. Finally, we employed the logistic regression model and calculated the receiver operating characteristic (ROC) curve. The pseudo-R2 was calculated to verify the model’s explanatory power, and the odds ratio (OR) was calculated. All p values < 0.05 were considered statistically significant. For the analyses, we used the statistical program Stata® version 17.

## Results

### Demographic and clinical characteristics of COVID-19 patients

Out of 184 COVID-19 patients admitted to the ICU, 177 (96.2%) were included in the study. We excluded seven patients due to incomplete medical records. The average age of the patients was 62.58 ± 14.39 years, with 61,58% being 60 years or older. The mortality rate was 61.58%, with higher rates among elderly patients (77.06%) and male patients (53.21%). The median length of stay in the ICU was 17.00 (1–166) days, ranging from 19.00 (1–166) days for survivors, to 17.00 (1–93) days for non-survivors.

Regarding comorbidities, 66.67% of patients had at least one comorbidity, with hypertension being the most common (44.63%), followed by diabetes (26.55%) and obesity (8.47%). No significant association was found between comorbidities and mortality.

As for supportive procedures, 76.27% of patients required mechanical ventilation, with a significant difference between the survivors (44.12%) and non-survivors (96.33%) groups (p <0.001). Almost all patients admitted to the ICU received antimicrobials (98.31%), with no significant difference between survivor and non-survivor groups (p = 0.286). More details about the demographic and clinical characteristics of the cohort can be seen in the supplementary data (S1–S3 Tables).

### Changes in hematological parameters in patients with severe COVID-19

The laboratory parameters showed no significant differences in hemoglobin, hematocrit, lymphocytes, monocytes, eosinophils, basophils, and platelet count between the survivor and non-survivor groups. However, non-survivors had significantly higher leukocyte (p<0.001) and neutrophil (p<0.001) counts, as shown in Table 2. Additionally, the inflammatory marker CRP was also significantly elevated (p = 0.048) in non-survivor patients.

**Table 2:** Demographic, clinical, and laboratory characteristics of ICU patients diagnosed with COVID-19.

Variables	Overall	Survivor	Non-survivor	p
**Number of patients**	177	68 (38.42%)	109 (61.58%)	
**Length of Stay, days (median)**	17.00 (1–166)	19.50 (1–166)	17.00 (1–93)	0.066
**Age, years (mean, SD)**	62.58 ± 14.39	55.65 ± 13.24	66.91 ± 13.40	<0.001*
**Age, (%)**				<0.001*
< 60	38.42	63.24	22.94	
≥ 60	61.58	36.76	77.06	
**Sex (%)**				0.465
Men	55.37	58.82	53.21	
Woman	44.63	41.18	46.79	
**Comorbidities**				
Systemic arterial hypertension (%)	44.63	39.71	47.71	0.298
Diabetes mellitus (%)	26.55	23.53	28.44	0.472
Obesity (%)	8.47	4.41	11.01	0.125
**Mechanical ventilation (%)**	76.27	44,12	96.33	<0.001*
**Use of Antimicrobials**	98.31	100.00	97.25	0.286
**Hematological Parameters**				
Hemoglobin (g/dL), median	12.7 (4.60–18.20)	12.7 (5.50–18.20)	12.5 (4.60–17.40)	0.391
Hematocrit (%), median	37.3 (14.3–52.0)	37.90 (16.6–52.6)	36.7 (14.3–52.00)	0.429
Leukocytes (x 10^3^/μL), mean (SD)	14.24 ± 7.38	11.83 ± 5.39	15.75 ± 8.06	<0.001*
Neutrophils (x 10^3^/μL), mean (SD)	13.33 ± 10.94	10.85 ± 8.05	14.88 ± 12.19	<0.001*
Lymphocytes (x 10^3^/μL), mean (SD)	0.97 ± 0.65	1.03 ± 0.72	0.93 ± 0.60	0.353
Monocytes (x 10^3^/μL), mean (SD)	0.59 ± 0.39	0.57 ± 0.34	0.60 ± 0.42	0.867
Eosinophils (x 10^3^/μL), median	0.008 (0–1.493)	0.007 (0–1.060)	0.008 (0–1.493)	0.835
Platelets (x 10^3^/μL), mean (SD)	236.26 ± 106.88	242.08 ± 96.46	232.62 ± 113.18	0.317
CRP (mg/L), mean (SD)	98.57 ± 75.14	82.84 ± 66.65	107.64 ± 75.14	0.049*

CRP: C-reactive protein.

To assess the immunological status of the patients, peripheral blood inflammatory parameters and immune cell subset ratios were analyzed. The NLR and CLR were significantly higher in the non-survivor group. However, there were no significant differences in LMR and PLR between survivors and non-survivors, as shown in Table 3. Details of laboratory parameters and peripheral blood immune cell subset ratios are provided in S4 and S5 Tables, respectively.

**Table 3:** Inflammatory parameters and immune cell subsets ratios in COVID-19 ICU patients.

Variable	Overall	Survivor	Non-survivor	p
**LMR**	2.49 ± 3.85	2.21 ± 1.38	2.66 ± 4.79	0.294
**PLR**	341.89 ± 249.18	331.01 ± 226.91	348.68 ± 262.92	0.648
**NLR**	19.44 ± 18.64	14.90 ± 16.13	22.28 ± 19.58	0.003*
**CLR**	0.14 ± 0.17	0.10 ± 0.16	0.16 ± 0.17	0.009*

LMR: lymphocyte-to-monocyte ratio; PLR: platelet-to-lymphocyte ratio; NLR: neutrophil-to-lymphocyte ratio; CLR: C-reactive protein-to-lymphocyte ratio.

### Parameters associated with COVID-19 mortality

Age, the requirement of MV, leukocytosis, neutrophilia, and an increase in CRP, NLR, and CLR showed a statistically significant association with the mortality of ICU patients with COVID-19 in the univariate analysis. However, in the multivariate logistic regression analysis, only the mechanical ventilation (OR 35.687, 95% CI: 11.084–114.898, p< 0.001) and the NLR (OR 1.026, 95% CI: 1.003–1.050, p = 0.028) parameters remained statistically associated with the outcome of death. The multivariate logistic regression analysis model used the reverse stepwise approach until all variables were significant (Table 4).

**Table 4:** Multivariate logistic regression analysis of risk factors for mortality in hospitalized patients with COVID-19 in ICU.

Variable	Odds Ratio	SE	p-value	Confidence Interval (95%)	
				Lower	Upper
MV	35.687	21.280	<0.001	11.084	114.898
NLR	1.026	0.012	0.028	1.003	1.050

SE: standard error; MV: mechanical ventilation; NLR: Neutrophil-to-lymphocyte ratio.

**Fig 1:**
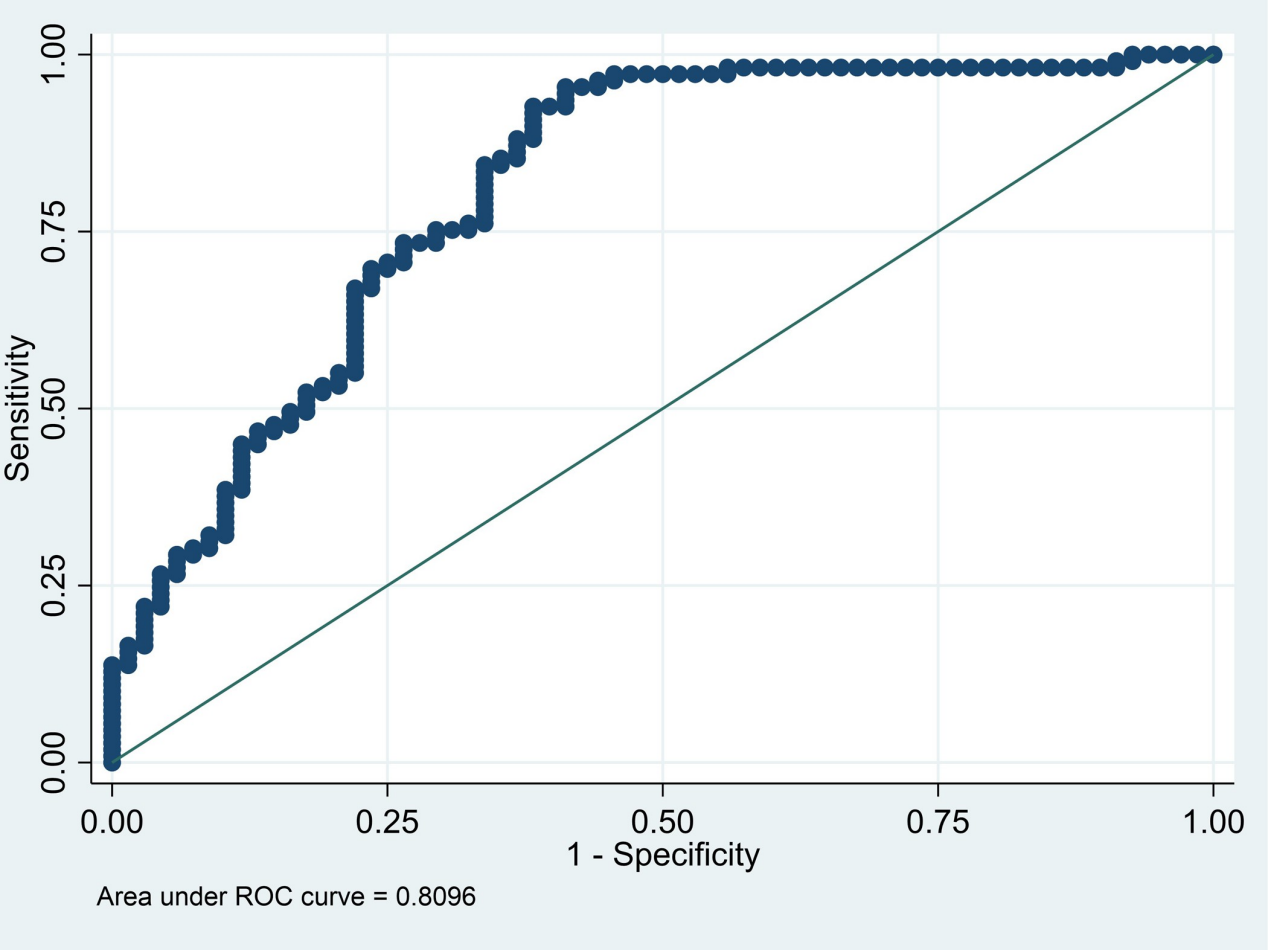
Receiver operating curve (ROC) multivariate logistic regression of the final model (MV and NLR).

The pseudo R2 of the final model (MV and NLR) was 0.304. The area under the curve was 0.8096, and the sensitivity and specificity were 96.33% and 54.41%, respectively (Fig 1).

## Discussion

In this cohort of severely ill COVID patients, we identified the NLR as the most critical hematological parameter to predict in-hospital mortality.

Elderly patients (≥ 60 years old) represented 77.06% of the non-survivor group in this study. In line with the literature, age showed to be significantly associated with mortality from COVID-19 in ICU patients (p < 0.001) [15–17]. According to Witkowski et al. (2022), it occurs in aging, a process of immunosenescence that affects the humoral and cellular immune functions, which reduces the effective response to pathogens. These changes could contribute to the increased severity and mortality related to COVID-19 infection in the elderly. In the same way, the immunosenescence process can be aggravated by SARS-CoV-2 (Severe Acute Respiratory Syndrome Coronavirus 2) infection. In addition, inflammatory aging contributes to an exacerbated inflammatory response and pro-inflammatory cytokine storm, promoting tissue damage and organ dysfunction [18, 19].

Regarding the comorbidities, hypertension (p = 0.298), diabetes mellitus (p = 0.472), or obesity (p = 0.125) alone showed no significant difference between the groups of survivors and non-survivors, differing from the results of other studies [15, 20]. However, the role of hypertension on the prognosis of COVID-19, for example, remains controversial in the literature [21].

The requirement for mechanical ventilation is a sign of severe COVID-19 and has been associated with death [3, 22–24]. Our results corroborate the literature data; almost all patients in the non-survival group (96.33%) received MV versus 44.12% in the survivors’ group. MV showed a positive association with the outcome of death in both univariate (p< 0.001) and multivariate logistic regression (OR 35.687, 95% CI: 11.084–114.898, p< 0.001). Patients who presented an intense inflammatory response associated with SARS-CoV-2 infection usually develop more severe symptoms and require ICU admission. The mortality rate of COVID-19 patients in the ICU can range from 0 to 84.6% [25]. In the present study, the overall mortality rate was 61.58%. It is essential to highlight that these data represented a period without vaccination or other proven effective treatment for COVID-19. According to epidemiological data, the period of the study comprised two waves of infection, the first with a prevalence of the B.1.195 variant (March and May 2020), and the second with a predominance of the variant P.1 (December 2020 and February 2021) [2, 26, 27].

Previous reports have demonstrated a significant increase in the white blood cells (WBC) and neutrophil count in severe COVID-19 patients. In contrast, the number of lymphocytes is reduced in acute infection [4, 28, 29]. Lymphopenia has been an important predictor of severity and mortality related to COVID-19 in patients older than 60 years [30]. In our study, leukocyte (p< 0.001) and neutrophil (p< 0.001) counts were significantly associated with mortality, being higher in non-survivors than in survivors.

We also observed a reduction in the number of lymphocytes (p = 0.353), but the difference was not significant between the groups. It is important to emphasize that these parameters have been used as a marker of severity among patients with mild, moderate, and severe COVID-19. Our study population is composed exclusively of critically ill patients, so some markers may not show a significant difference.

As lymphocytopenia, neutrophilia is a biomarker of acute infection. Neutrophils play a crucial role in the innate immune response, and the increase in their blood count has been associated with adverse outcomes in COVID-19 patients [31]. In the present study, neutrophil counts were statistically higher in the non-survivor group and were positively associated with mortality (p< 0.001).

In our data, CRP was another inflammatory biomarker positively correlated with patient mortality (p = 0.049). CRP is a non-specific acute-phase inflammation biomarker. Other authors also reported increased CRP in COVID-19 patients [31]. Jemaa et al. (2022) explored the relationship between CRP and the severity of COVID-19 and observed a significant increase associated with ICU admission and ICU mortality. Additionally, CRP levels were correlated to WBC and neutrophil counts in non-ICU and ICU patients [32].

Impaired blood coagulation function is observed in critical COVID-19 patients. Reduction in platelet blood count has been related to withdrawal from circulation by disseminated thrombotic events [33]. In the present study, the mean platelet count was slightly smaller in non-survivor patients (p = 0.317), disagreeing with other studies [34, 35]. However, this corroborated the study of Jemaa et al. (2022), which compared platelet count in ICU survivors and non-survivors [32].

In addition to the effects on circulating immune cells, SARS-CoV-2 infection also affects the production of blood cells by impairing hematopoiesis. The expansion of immature and dysfunctional neutrophils, accompanied by increased erythroid precursors in the circulation, is compatible with infection-induced stress hematopoiesis [36, 37].

It is possible to calculate inflammation markers, such as NLR, LMR, PLR, and CLR, that reflect systemic inflammatory response through routine laboratory parameters. The predictive role of these indices in the clinical severity of patients with COVID-19 has been primarily evaluated [38–41].

The predictive role of the LMR is still controversial in the literature, however, our results were consistent with Jemaa, et al. (2022), which showed no correlation between this parameter and severe COVID-19 [32].

Our results demonstrated that increases in NLR (p = 0.003) and CLR (p = 0.009) are associated with COVID-19 mortality in ICU patients. Still, only NLR remained a risk factor in the multivariate logistic regression model (OR 1.026, 95% CI: 1.003–1.050, p = 0.028).

Several studies have verified the role of the NLR in distinguishing non-severe and severe cases of COVID-19, including ICU admission. However, data evaluating the predictive value of the NLR to critically ill patients are extremely scarce in the literature.

Gholinataj Jelodar et al. (2023), in a cross-sectional study, evaluated the NLR parameter of ICU COVID-19 patients and demonstrated a significative difference between the groups of survivors and non-survivors. NLR was a risk factor in the multivariate logistic regression (OR 1.045, 95% CI: 1.012–1.079, p = 0.007) [42].

Farias et al. (2022) evaluated the association of leukocyte biomarkers, calculated in the emergency department, with the COVID-19 severity and mortality, in a cohort retrospective study, which includes 1,535 patients. They observed that NLR, MLR, and PLR had significant correlations with mortality, but only NLR was independently associated with both outcomes on multivariate analysis [43].

According to a recent meta-analysis conducted by Cheng et al. (2021), which analyzed 17 studies and 7,049 COVID-19 patients, it was found that the NLR (neutrophil-to-lymphocyte ratio) was significantly higher in patients with severe COVID-19 compared to those with non-severe COVID-19 [44]. This study reinforces that the initial NLR can be a reliable predictor of severe COVID-19.

In a study conducted by Asghar et al. (2022), they analyzed data from 1,000 COVID-19 patients who were hospitalized. The study found that both NLR and dNLR (which is calculated by dividing the absolute neutrophil count by the total leucocyte count minus the absolute neutrophil count) are reliable and sensitive markers for predicting the in-hospital outcomes of patients with COVID-19 [13]. Although, the NLR parameter has been more largely evaluated in the literature.

Khalid et al. (2022) compared hematological parameters between patients with COVID-19 and a control group of healthy individuals and observed that the absolute count of lymphocytes, PLR, and NLR was significantly higher in cases of severe disease [35].

Kosidło et al. (2023), in a recent literature review, reinforce the role of NLR as a predictor of the severity of inflammation in the course of COVID-19. On the other hand, it demonstrates the existence of discrepancies in the results involving the role of LMR [45].

As mentioned before, although several studies have addressed inflammatory parameters as predictors of severity and mortality [29, 46, 47], the role of these indices in distinguishing the outcome of death among critically ill ICU patients has rarely been investigated in previous research. Therefore, our results are an important contribution to establishing NLR as a predictor of mortality among critically ill patients admitted to the ICU.

In the current scenario, with a spread vaccination of the world population, the consolidation of hematological prognostic biomarkers remains important for any prospective targeted intervention in vulnerable patients, as the immunocompromised. In addition, the knowledge acquired with COVID-19 will undoubtedly serve as a basis for facing possible new viruses.

The main limitation of the study was the retrospective and unicentric design. Hence, confounding factors may have also affected the outcomes. In addition, the blood parameters assessed were related to the admission period, and changes throughout the hospital stay could have other clinical implications. Further studies, with multicenter and prospective manner, will be important to corroborate the role of leukocyte ratios in discriminating the risk of death in patients with severe COVID-19 admitted to the ICU.

## Conclusion

Mechanical ventilation and neutrophil to lymphocyte ratio were shown to be independent predictors of mortality in patients with severe COVID-19 admitted to the ICU. While the need for mechanical ventilation is a parameter observed throughout the hospital stay, the initial NLR can be a prior risk stratification tool to establish priorities and timely clinical intervention. NLR parameter has a potential clinical implication distinguishing the patients who could benefit from pharmacotherapy with anti-inflammatories such as corticoids and tocilizumab, an IL-6 antagonist.

## Supporting information

S1 Table Characteristics of patients admitted to the ICU diagnosed with COVID-19, according to gender. (DOCX)

S2 Table Demographic and clinical characteristics of ICU patients with COVID-19. (DOCX)

S3 Table Clinical characteristics of ICU patients diagnosed with COVID-19. (DOCX)

S4 Table Laboratory characteristics of ICU patients diagnosed with COVID-19. (DOCX)

S5 Table Inflammatory parameters and immune cell subsets ratios in COVID-19 ICU patients. (DOCX)

## References

[1] Huang C, Wang Y, Li X, Ren L, Zhao J, Hu Y, et al. Clinical features of patients infected with 2019 novel coronavirus in Wuhan, China. Lancet. 2020 Feb 15;395(10223):497–506. doi: 10.1016/S0140-6736(20)30183-5 31986264 PMC7159299

[2] Naveca FG, Nascimento V, De Souza VC, Corado ADL, Nascimento F, Silva G, et al. COVID-19 in Amazonas, Brazil, was driven by the persistence of endemic lineages and P.1 emergence. Nat Med. 2021 Jul;27(7):1230–8. doi: 10.1038/s41591-021-01378-7 34035535

[3] Chen N, Zhou M, Dong X, Qu J, Gong F, Han Y, et al. Epidemiological and clinical characteristics of 99 cases of 2019 novel coronavirus pneumonia in Wuhan, China: a descriptive study. Lancet. 2020 Feb 15;395(10223):507–13. doi: 10.1016/S0140-6736(20)30211-7 32007143 PMC7135076

[4] Qin C, Zhou L, Hu Z, Zhang S, Yang S, Tao Y, et al. Dysregulation of Immune Response in Patients with Coronavirus 2019 (COVID-19) in Wuhan, China. Clin Infect Dis. 2020 Jul 28;71(15):762–8. doi: 10.1093/cid/ciaa248 32161940 PMC7108125

[5] Mehta P, McAuley DF, Brown M, Sanchez E, Tattersall RS, Manson JJ, et al. COVID-19: consider cytokine storm syndromes and immunosuppression. Lancet. 2020 Mar 28;395(10229):1033–4. doi: 10.1016/S0140-6736(20)30628-0 32192578 PMC7270045

[6] Keskin M, Burcak Polat S, Ateş I, Izdeş S, Rahmet Güner H, Topaloğlu O, et al. Are neutrophil-to-lymphocyte ratios and large unstained cells different in hospitalized COVID-19 PCR-positive patients with and without diabetes mellitus? Eur Rev Pharmacol Sci. 2022 Aug;26(16):5963–70. doi: 10.26355/eurrev_202208_29537 36066173

[7] Diamond MS, Kanneganti TD. Innate immunity: the first line of defense against SARS-CoV-2. Nat Immunol. 2022 Feb;23(2):165–76. doi: 10.1038/s41590-021-01091-0 35105981 PMC8935980

[8] Tong X, Cheng A, Yuan X, Zhong X, Wang H, Zhou W, et al. Characteristics of peripheral white blood cells in COVID-19 patients revealed by a retrospective cohort study. BMC Infect Dis. 2021 Dec 9;21(1):1236. doi: 10.1186/s12879-021-06899-7 34886793 PMC8655490

[9] Zhao L, Zhang YP, Yang X, Liu X. Eosinopenia is associated with greater severity in patients with coronavirus disease 2019. Allergy. 2021 Feb;76(2):562–4. doi: 10.1111/all.14455 32544252 PMC7323424

[10] Glickman JW, Pavel AB, Guttman‐Yassky E, Miller RL. The role of circulating eosinophils on COVID‐19 mortality varies by race/ethnicity. Allergy. 2021 Mar;76(3):925–7. doi: 10.1111/all.14708 33319360 PMC8290320

[11] Chelariu AC, Coman AE, Lionte C, Gorciac V, Sorodoc V, Haliga RE, et al. The Value of Early and Follow-Up Elevated Scores Based on Peripheral Complete Blood Cell Count for Predicting Adverse Outcomes in COVID-19 Patients. J Pers Med. 2022 Dec 9;12(12):2037. doi: 10.3390/jpm12122037 36556258 PMC9781715

[12] Yang X, Yu Y, Xu J, Shu H, Xia J, Liu H, et al. Clinical course and outcomes of critically ill patients with SARS-CoV-2 pneumonia in Wuhan, China: a single-centered, retrospective, observational study. Lancet Respir Med. 2020 May;8(5):475–81. doi: 10.1016/S2213-2600(20)30079-5 32105632 PMC7102538

[13] Asghar MS, Akram M, Yasmin F, Najeeb H, Naeem U, Gaddam M, et al. Comparative analysis of neutrophil to lymphocyte ratio and derived neutrophil to lymphocyte ratio with respect to outcomes of in-hospital coronavirus disease 2019 patients: A retrospective study. Front Med. 2022 Jul 22;9:951556. doi: 10.3389/fmed.2022.951556 35935776 PMC9354523

[14] Piovani D, Tsantes AG, Bonovas S. Prognostic Role of Neutrophil-to-Lymphocyte Ratio in Patients with COVID-19. J Clin Med. 2022 Aug 11;11(16):4688. doi: 10.3390/jcm11164688 36012928 PMC9410484

[15] Baguma S, Okot C, Alema NO, Apiyo P, Layet P, Acullu D, et al. Factors Associated With Mortality Among the COVID-19 Patients Treated at Gulu Regional Referral Hospital: A Retrospective Study. Front Public Health. 2022 Apr 11;10:841906. doi: 10.3389/fpubh.2022.841906 35480594 PMC9035511

[16] Wei C, Liu Y, Liu Y, Zhang K, Su D, Zhong M, et al. Clinical characteristics and manifestations in older patients with COVID-19. BMC Geriatr. 2020 Oct 8;20(1):395. doi: 10.1186/s12877-020-01811-5 33032534 PMC7542569

[17] Wong CKH, Wong JYH, Tang EHM, Au CH, Wai AKC. Clinical presentations, laboratory and radiological findings, and treatments for 11,028 COVID-19 patients: a systematic review and meta-analysis. Sci Rep. 2020 Nov 13;10(1):19765. doi: 10.1038/s41598-020-74988-9 33188232 PMC7666204

[18] Witkowski JM, Fulop T, Bryl E. Immunosenescence and COVID-19. Mech Ageing Dev. 2022 Jun;204:111672. doi: 10.1016/j.mad.2022.111672 35378106 PMC8975602

[19] Reyna-Villasmil E, Caponcello MG, Maldonado N, Olivares P, Caroccia N, Bonazzetti C, et al. Association of Patients’ Epidemiological Characteristics and Comorbidities with Severity and Related Mortality Risk of SARS-CoV-2 Infection: Results of an Umbrella Systematic Review and Meta-Analysis. Biomedicines. 2022 Sep 29;10(10):2437. doi: 10.3390/biomedicines10102437 36289699 PMC9598435

[20] Baradaran A, Ebrahimzadeh MH, Baradaran A, Kachooei AR. Prevalence of Comorbidities in COVID-19 Patients: A Systematic Review and Meta-Analysis. Arch Bone Jt Surg. 2020 Apr;8(Suppl 1):247–55. doi: 10.22038/abjs.2020.47754.2346 32733980 PMC7296605

[21] Tadic M, Saeed S, Grassi G, Taddei S, Mancia G, Cuspidi C. Hypertension and COVID-19: Ongoing Controversies. Front Cardiovasc Med. 2021 Feb 17;8:639222. doi: 10.3389/fcvm.2021.639222 33681308 PMC7925389

[22] Badin RC, Amorim RLOD, Aguila A, Manaças LRA. Clinical and pharmacological factors associated with mortality in patients with COVID-19 in a high complexity hospital in Manaus: A retrospective study. Liu BM, editor. PLoS One. 2023 Feb 10;18(2):e0280891. doi: 10.1371/journal.pone.0280891 36763604 PMC9916623

[23] Bahl A, Van Baalen MN, Ortiz L, Chen NW, Todd C, Milad M, et al. Early predictors of in-hospital mortality in patients with COVID-19 in a large American cohort. Intern Emerg Med. 2020 Nov;15(8):1485–99. doi: 10.1007/s11739-020-02509-7 32970246 PMC7512216

[24] Zhou F, Yu T, Du R, Fan G, Liu Y, Liu Z, et al. Clinical course and risk factors for mortality of adult inpatients with COVID-19 in Wuhan, China: a retrospective cohort study. Lancet. 2020 Mar 28;395(10229):1054–62. doi: 10.1016/S0140-6736(20)30566-3 32171076 PMC7270627

[25] Armstrong RA, Kane AD, Cook TM. Outcomes from intensive care in patients with COVID‐19: a systematic review and meta‐analysis of observational studies. Anaesthesia. 2020 Oct;75(10):1340–9. doi: 10.1111/anae.15201 32602561

[26] Buss LF, Prete CA, Abrahim CMM, Mendrone A, Salomon T, De Almeida-Neto C, et al. Three-quarters attack rate of SARS-CoV-2 in the Brazilian Amazon during a largely unmitigated epidemic. Science. 2021 Jan 15;371(6526):288–92. doi: 10.1126/science.abe9728 33293339 PMC7857406

[27] Coutinho RM, Marquitti FMD, Ferreira LS, Borges ME, da Silva RLP, Canton O, et al. Model-based estimation of transmissibility and reinfection of SARS-CoV-2 P.1 variant. Commun Med (Lond). 2021 Nov 15;1:48. doi: 10.1038/s43856-021-00048-6 35602219 PMC9053218

[28] Tan L, Wang Q, Zhang D, Ding J, Huang Q, Tang YQ, et al. Lymphopenia predicts disease severity of COVID-19: a descriptive and predictive study. Signal Transduct Target Ther. 2020 Mar 27;5(1):33. doi: 10.1038/s41392-020-0148-4 32296069 PMC7100419

[29] Idiz UO, Yurttas TT, Degirmencioglu S, Orhan B, Erdogan E, Sevik H, et al. Immunophenotyping of lymphocytes and monocytes and the status of cytokines in the clinical course of Covid‐19 patients. J Med Virol. 2022 Oct;94(10):4744–53. doi: 10.1002/jmv.27917 35667877 PMC9348494

[30] Zhou Y, Fu B, Zheng X, Wang D, Zhao C, Qi Y, et al. Pathogenic T-cells and inflammatory monocytes incite inflammatory storms in severe COVID-19 patients. Natl Sci Rev. 2020 Jun;7(6):998–1002. doi: 10.1093/nsr/nwaa041 34676125 PMC7108005

[31] Henry BM, De Oliveira MHS, Benoit S, Plebani M, Lippi G. Hematologic, biochemical and immune biomarker abnormalities associated with severe illness and mortality in coronavirus disease 2019 (COVID-19): a meta-analysis. Clin Chem Lab Med. 2020 Jun 25;58(7):1021–8. doi: 10.1515/cclm-2020-0369 32286245

[32] Ben Jemaa A, Salhi N, Ben Othmen M, Ben Ali H, Guissouma J, Ghadhoune H, et al. Evaluation of individual and combined NLR, LMR and CLR ratio for prognosis disease severity and outcomes in patients with COVID-19. Int Immunopharmacol. 2022 Aug;109:108781. doi: 10.1016/j.intimp.2022.108781 35461157 PMC9015974

[33] Maquet J, Lafaurie M, Sommet A, Moulis G. Thrombocytopenia is independently associated with poor outcome in patients hospitalized for COVID‐19. Br J Haematol. 2020 Sep; 190 (5):e276–9. doi: 10.1111/bjh.16950 32557535 PMC7323390

[34] Ertekin B, Yortanlı M, Özelbaykal O, Doğru A, Girişgin AS, Acar T. The Relationship between Routine Blood Parameters and the Prognosis of COVID-19 Patients in the Emergency Department. Emerg Med Int. 2021 Dec 1;2021:7489675. doi: 10.1155/2021/7489675 34868686 PMC8633851

[35] Khalid AM a. M, Suliman AM, Abdallah EI, Abakar M a. A, Elbasheir MM, Muddathir AM, et al. Influence of COVID-19 on lymphocyte and platelet parameters among patients admitted to intensive care unit and emergency. Eur Rev Med Pharmacol Sci. 2022 Apr;26(7):2579–85. 10.26355/eurrev_202204_28495 35442473

[36] Elahi S. Hematopoietic responses to SARS-CoV-2 infection. Cell Mol Life Sci. 2022 Mar 13;79(3):187. doi: 10.1007/s00018-022-04220-6 35284964 PMC8918078

[37] Balandrán JC, Zamora-Herrera G, Romo-Rodríguez R, Pelayo R. Emergency Hematopoiesis in the Pathobiology of COVID-19: The Dark Side of an Early Innate Protective Mechanism. J Interferon Cytokine Res. 2022 Aug 1;42(8):393–405. doi: 10.1089/jir.2022.0028 35675647

[38] Malik P, Patel U, Mehta D, Patel N, Kelkar R, Akrmah M, et al. Biomarkers and outcomes of COVID-19 hospitalisations: systematic review and meta-analysis. BMJ Evid Based Med. 2021 Jun;26(3):107–8. doi: 10.1136/bmjebm-2020-111536 32934000 PMC7493072

[39] Lagunas‐Rangel FA. Neutrophil‐to‐lymphocyte ratio and lymphocyte‐to‐C‐reactive protein ratio in patients with severe coronavirus disease 2019 (COVID‐19): A meta‐analysis. J Med Virol. 2020 Oct;92(10):1733–4. doi: 10.1002/jmv.25819 32242950 PMC7228336

[40] Danwang C, Endomba FT, Nkeck JR, Wouna DLA, Robert A, Noubiap JJ. A meta-analysis of potential biomarkers associated with severity of coronavirus disease 2019 (COVID-19). Biomark Res. 2020 Aug 31;8(1):37. doi: 10.1186/s40364-020-00217-0 32879731 PMC7456766

[41] Chaudhary R, Garg J, Houghton DE, Murad MH, Kondur A, Chaudhary R, et al. Thromboinflammatory Biomarkers in COVID-19: Systematic Review and Meta-analysis of 17,052 Patients. Mayo Clin Proc Innov Qual Outcomes. 2021 Apr;5(2):388–402. doi: 10.1016/j.mayocpiqo.2021.01.009 33585800 PMC7869679

[42] Gholinataj Jelodar M, Rafieian S, Allah Dini A, Khalaj F, Zare S, Dehghanpour H, et al. Analyzing Trends in Demographic, Laboratory, Imaging, and Clinical Outcomes of ICU-Hospitalized COVID-19 Patients. Can J Infect Dis Med Microbiol. 2023 May; 29;2023:3081660. doi: 10.1155/2023/3081660 37283598 PMC10241583

[43] Farias JP, Silva PPCE, Codes L, Vinhaes D, Amorim AP, D’Oliveira RC, et al. Leukocyte ratios are useful early predictors for adverse outcomes of COVID-19 infection. Rev Inst Med trop S Paulo. 2022 Nov; 14;64:e73. doi: 10.1590/S1678-9946202264073 36383895 PMC9673132

[44] Cheng J, Ma A, Yang J, Dong M, Liao X, Kang Y. The neutrophil to lymphocyte ratio is an independent predictor for severe COVID-19: Evidence from a multicenter case-control study and meta-analyses. Wien Klin Wochenschr. 2021 Sep;133(17–18):882–91. 34342712 10.1007/s00508-021-01917-9 PMC8329905

[45] Kosidło JW, Wolszczak-Biedrzycka B, Matowicka-Karna J, Dymicka-Piekarska V, Dorf J. Clinical significance and diagnostic utility of NLR, LMR, PLR and SII in the course of COVID-19: a literature review. J Inflamm Res. 2023 Feb 11; 16:539–62. doi: 10.2147/JIR.S395331 36818192 PMC9930576

[46] Martins PM, Gomes TLN, Franco EP, Vieira LL, Pimentel GD. High neutrophil‐to‐lymphocyte ratio at intensive care unit admission is associated with nutrition risk in patients with COVID‐19. J Parenter Enteral Nutr. 2022 Aug;46(6):1441–8. doi: 10.1002/jpen.2318 34961953 PMC9015430

[47] Agarwal S. Neutrophil-Lymphocyte Ratio Predicting Case Severity in SARS-CoV-2 Infection: A Review. Cureus 2022 Sep 29; 14(9): e29760. doi: 10.7759/cureus.29760 36187170 PMC9521818

