# Supplementary material for: Evaluation of hematological changes and immune response biomarkers as a prognostic factor in critical patients with COVID-19
Source: PLoS One. 2024 Feb 29;19(2):e0297490. doi: 10.1371/journal.pone.0297490 (PMC10903867; doi:10.1371/journal.pone.0297490)
Supplement: S1 Table — (DOCX) [file pone.0297490.s001.docx]

**Supporting information**

S1 Table. Characteristics of patients admitted to the ICU diagnosed with COVID-19, according to gender.

| **Sex (n)/%** |  | **Man (98)** | **Woman (79)** | **p- value** |
| --- | --- | --- | --- | --- |
| Age |  | 61.67 ± 14.08 | 63.72 ± 14.79 | 0.385 |
| MV | Yes | 74.49 | 78.48 | 0.535 |
|  | No | 25.51 | 21.52 |  |
| Comorbidities (n) | Yes | 66.33 | 67.09 | 0.915 |
|  | No | 33.67 | 32.91 |  |
| HAS | Yes | 46.94 | 41.77 | 0.492 |
|  | No | 53.06 | 58.23 |  |
| DM | Yes | 28.57 | 24.05 | 0.498 |
|  | No | 71.43 | 75.95 |  |
| Obesity | Yes | 7.14 | 10.13 | 0.479 |
|  | No | 92.86 | 89.87 |  |
| Corticosteroids (n) | Yes | 96.94 | 96.20 | 0.552 |
|  | No | 3.06 | 3.80 |  |
| Antimicrobials (n) | Yes | 97.96 | 98.73 | 0.581 |
|  | No | 2.04 | 1.27 |  |
