# Supplementary material for: Evaluation of hematological changes and immune response biomarkers as a prognostic factor in critical patients with COVID-19
Source: PLoS One. 2024 Feb 29;19(2):e0297490. doi: 10.1371/journal.pone.0297490 (PMC10903867; doi:10.1371/journal.pone.0297490)
Supplement: S2 Table — (DOCX) [file pone.0297490.s002.docx]

**Supporting information**

**S2 Table. Demographic and clinical characteristics of ICU patients diagnosed with COVID-19.**

| **Sample** | **Gender** | **Elderly** | **Length of stay** | **Comorbidity** | **Obesity** |
| --- | --- | --- | --- | --- | --- |
| 1 CV | Female | Yes | 37 | Yes | No |
| 2 CV | Female | No | 27 | No | No |
| 3 CV | Female | No | 70 | Yes | No |
| 4 CV | Male | No | 14 | Yes | Yes |
| 5 CV | Female | Yes | 11 | Yes | Yes |
| 6 CV | Female | Yes | 45 | Yes | No |
| 7 CV | Female | Yes | 11 | Yes | Yes |
| 8 CV | Male | Yes | 41 | Yes | Yes |
| 9 CV | Male | Yes | 3 | Yes | No |
| 10 CV | Female | Yes | 11 | No | No |
| 11 CV | Male | Yes | 19 | Yes | No |
| 12 CV | Male | No | 11 | No | No |
| 13 CV | Female | Yes | 22 | No | No |
| 14 CV | Female | Yes | 8 | Yes | No |
| 15 CV | Female | Yes | 29 | Yes | No |
| 16 CV | Female | Yes | 33 | Yes | No |
| 17 CV | Female | Yes | 166 | Yes | No |
| 18 CV | Female | Yes | 17 | Yes | Yes |
| 19 CV | Female | Yes | 20 | Yes | No |
| 20 CV | Male | Yes | 43 | Yes | No |
| 21 CV | Male | No | 32 | Yes | No |
| 22 CV | Male | No | 21 | No | No |
| 23 CV | Male | Yes | 11 | No | No |
| 24 CV | Male | Yes | 7 | Yes | No |
| 25 CV | Male | Yes | 38 | Yes | No |
| 26 CV | Male | Yes | 9 | No | No |
| 27 CV | Male | No | 31 | Yes | No |
| 28 CV | Female | Yes | 44 | Yes | No |
| 29 CV | Male | No | 15 | No | No |
| 30 CV | Female | Yes | 1 | No | No |
| 31 CV | Female | No | 19 | Yes | No |
| 32 CV | Male | Yes | 17 | Yes | No |
| 33 CV | Female | Yes | 17 | Yes | Yes |
| 34 CV | Male | No | 25 | Yes | No |
| 35 CV | Female | No | 23 | Yes | No |
| 36 CV | Female | Yes | 15 | Yes | No |
| 37 CV | Male | No | 22 | Yes | No |
| 38 CV | Male | No | 9 | No | No |
| 39 CV | Female | Yes | 11 | Yes | No |
| 40 CV | Female | No | 13 | Yes | No |
| 41 CV | Female | No | 93 | Yes | No |
| 42 CV | Female | Yes | 31 | Yes | No |
| 43 CV | Male | Yes | 26 | Yes | No |
| 44 CV | Female | Yes | 56 | Yes | No |
| 45 CV | Male | Yes | 21 | Yes | No |
| 46 CV | Female | Yes | 56 | Yes | Yes |
| 47 CV | Male | Yes | 9 | Yes | No |
| 48 CV | Male | Yes | 27 | Yes | No |
| 49 CV | Female | Yes | 21 | No | No |
| 50 CV | Female | No | 39 | No | No |
| 51 CV | Male | Yes | 21 | No | No |
| 52 CV | Male | Yes | 7 | Yes | No |
| 53 CV | Female | Yes | 25 | Yes | Yes |
| 54 CV | Male | Yes | 27 | Yes | No |
| 55 CV | Male | Yes | 18 | Yes | No |
| 56 CV | Male | Yes | 8 | Yes | No |
| 57 CV | Female | Yes | 8 | No | No |
| 58 CV | Male | No | 52 | No | No |
| 59 CV | Male | Yes | 9 | Yes | No |
| 60 CV | Male | Yes | 8 | Yes | No |
| 61 CV | Female | No | 21 | Yes | Yes |
| 62 CV | Female | No | 15 | No | No |
| 63 CV | Female | Yes | 26 | No | No |
| 64 CV | Female | No | 15 | Yes | No |
| 65 CV | Male | Yes | 20 | Yes | No |
| 66 CV | Male | Yes | 11 | Yes | No |
| 67 CV | Male | Yes | 41 | Yes | No |
| 68 CV | Female | No | 32 | No | No |
| 69 CV | Female | Yes | 69 | Yes | No |
| 70 CV | Female | Yes | 63 | No | No |
| 71 CV | Male | No | 24 | Yes | No |
| 72 CV | Male | No | 8 | Yes | No |
| 73 CV | Male | Yes | 22 | Yes | No |
| 74 CV | Female | Yes | 2 | No | No |
| 75 CV | Male | No | 23 | No | No |
| 76 CV | Male | Yes | 39 | Yes | No |
| 77 CV | Male | No | 10 | Yes | Yes |
| 78 CV | Female | Yes | 30 | Yes | No |
| 79 CV | Female | No | 11 | No | No |
| 80 CV | Female | No | 7 | Yes | No |
| 81 CV | Male | Yes | 6 | Yes | Yes |
| 82 CV | Male | No | 43 | No | No |
| 83 CV | Male | No | 22 | Yes | No |
| 84 CV | Female | Yes | 2 | Yes | No |
| 85 CV | Female | Yes | 12 | Yes | No |
| 86 CV | Male | Yes | 63 | Yes | No |
| 87 CV | Female | No | 16 | No | No |
| 88 CV | Male | Yes | 41 | Yes | No |
| 89 CV | Male | Yes | 33 | No | No |
| 90 CV | Male | Yes | 18 | Yes | No |
| 91 CV | Male | Yes | 4 | Yes | No |
| 92 CV | Male | No | 8 | Yes | No |
| 93 CV | Female | Yes | 14 | Yes | No |
| 94 CV | Male | Yes | 1 | No | No |
| 95 CV | Male | Yes | 12 | Yes | No |
| 96 CV | Female | Yes | 13 | Yes | No |
| 97 CV | Male | No | 9 | Yes | No |
| 98 CV | Female | No | 11 | Yes | No |
| 99 CV | Female | Yes | 21 | No | No |
| 100 CV | Female | Yes | 16 | Yes | No |
| 101 CV | Male | Yes | 7 | No | No |
| 102 CV | Male | No | 24 | Yes | No |
| 103 CV | Female | Yes | 17 | Yes | No |
| 104 CV | Female | No | 9 | Yes | No |
| 105 CV | Female | No | 3 | Yes | No |
| 106 CV | Female | Yes | 12 | Yes | No |
| 107 CV | Female | No | 13 | Yes | No |
| 108 CV | Male | Yes | 18 | No | No |
| 109 CV | Male | Yes | 1 | No | No |
| 110 CV | Male | No | 15 | No | No |
| 111 CV | Female | Yes | 18 | Yes | No |
| 112 CV | Male | Yes | 4 | Yes | No |
| 113 CV | Male | Yes | 12 | No | No |
| 114 CV | Male | No | 7 | No | No |
| 115 CV | Male | No | 20 | No | No |
| 116 CV | Male | No | 48 | Yes | No |
| 117 CV | Female | Yes | 17 | Yes | Yes |
| 118 CV | Male | Yes | 54 | Yes | No |
| 119 CV | Male | No | 8 | Yes | No |
| 120 CV | Male | Yes | 2 | Yes | No |
| 121 CV | Male | Yes | 0 | No | No |
| 122 CV | Male | Yes | 14 | Yes | No |
| 123 CV | Male | No | 17 | Yes | No |
| 124 CV | Male | No | 32 | Yes | No |
| 125 CV | Male | Yes | 30 | Yes | Yes |
| 126 CV | Female | No | 33 | Yes | No |
| 127 CV | Male | Yes | 15 | Yes | No |
| 128 CV | Female | Yes | 5 | Yes | No |
| 129 CV | Male | No | 12 | No | No |
| 130 CV | Female | Yes | 2 | Yes | No |
| 131 CV | Female | No | 50 | No | No |
| 132 CV | Female | No | 11 | No | No |
| 133 CV | Female | No | 12 | Yes | No |
| 134 CV | Female | No | 28 | No | No |
| 135 CV | Male | Yes | 14 | Yes | No |
| 136 CV | Male | Yes | 12 | Yes | No |
| 137 CV | Male | No | 16 | Yes | Yes |
| 138 CV | Male | No | 49 | Yes | No |
| 139 CV | Male | Yes | 30 | Yes | No |
| 140 CV | Male | Yes | 12 | No | No |
| 141 CV | Female | Yes | 16 | Yes | No |
| 142 CV | Male | No | 9 | No | No |
| 143 CV | Female | No | 10 | No | No |
| 144 CV | Male | No | 41 | Yes | No |
| 145 CV | Male | Yes | 13 | No | No |
| 146 CV | Male | No | 8 | Yes | Yes |
| 147 CV | Female | Yes | 23 | No | No |
| 148 CV | Male | No | 7 | No | No |
| 149 CV | Male | Yes | 29 | Yes | No |
| 150 CV | Male | Yes | 8 | No | No |
| 151 CV | Male | No | 18 | Yes | No |
| 152 CV | Female | No | 27 | No | No |
| 153 CV | Male | Yes | 18 | Yes | No |
| 154 CV | Female | No | 4 | No | No |
| 155 CV | Female | Yes | 37 | Yes | No |
| 156 CV | Male | Yes | 3 | Yes | No |
| 157 CV | Male | No | 17 | No | No |
| 158 CV | Female | Yes | 20 | Yes | No |
| 159 CV | Female | Yes | 2 | No | No |
| 160 CV | Male | No | 15 | No | No |
| 161 CV | Male | No | 1 | No | No |
| 162 CV | Male | Yes | 28 | Yes | No |
| 163 CV | Male | Yes | 30 | Yes | No |
| 164 CV | Female | Yes | 24 | No | No |
| 165 CV | Female | Yes | 5 | Yes | No |
| 166 CV | Female | No | 69 | No | No |
| 167 CV | Male | Yes | 30 | No | No |
| 168 CV | Male | No | 36 | No | No |
| 169 CV | Female | No | 122 | Yes | No |
| 170 CV | Male | Yes | 7 | Yes | No |
| 171 CV | Female | Yes | 44 | Yes | No |
| 172 CV | Female | No | 25 | No | No |
| 173 CV | Female | Yes | 15 | Yes | No |
| 174 CV | Male | Yes | 50 | Yes | No |
| 175 CV | Male | No | 24 | No | No |
| 176 CV | Male | Yes | 4 | No | No |
| 177 CV | Female | Yes | 45 | Yes | No |
