# Supplementary material for: Evaluation of hematological changes and immune response biomarkers as a prognostic factor in critical patients with COVID-19
Source: PLoS One. 2024 Feb 29;19(2):e0297490. doi: 10.1371/journal.pone.0297490 (PMC10903867; doi:10.1371/journal.pone.0297490)
Supplement: S3 Table — (DOCX) [file pone.0297490.s003.docx]

**Supporting information**

**S3 Table. Clinical characteristics of ICU patients diagnosed with COVID-19.**

| **Sample** | **Hypertension** | **Mechamical ventilation** | **Antimicrobials** | **Death** |
| --- | --- | --- | --- | --- |
| 1 CV | Yes | Yes | Yes | Yes |
| 2 CV | No | Yes | Yes | No |
| 3 CV | No | Yes | Yes | No |
| 4 CV | No | Yes | Yes | Yes |
| 5 CV | No | Yes | Yes | Yes |
| 6 CV | Yes | Yes | Yes | Yes |
| 7 CV | No | Yes | Yes | Yes |
| 8 CV | Yes | Yes | Yes | No |
| 9 CV | No | Yes | Yes | Yes |
| 10 CV | No | Yes | Yes | Yes |
| 11 CV | Yes | No | Yes | No |
| 12 CV | No | No | Yes | No |
| 13 CV | No | Yes | Yes | Yes |
| 14 CV | Yes | Yes | Yes | Yes |
| 15 CV | No | Yes | Yes | Yes |
| 16 CV | Yes | Yes | Yes | Yes |
| 17 CV | Yes | Yes | Yes | No |
| 18 CV | Yes | Yes | Yes | Yes |
| 19 CV | No | No | Yes | No |
| 20 CV | Yes | Yes | Yes | Yes |
| 21 CV | No | Yes | Yes | Yes |
| 22 CV | No | No | Yes | No |
| 23 CV | No | No | Yes | No |
| 24 CV | No | Yes | Yes | Yes |
| 25 CV | Yes | Yes | Yes | Yes |
| 26 CV | No | Yes | Yes | Yes |
| 27 CV | Yes | Yes | Yes | Yes |
| 28 CV | Yes | Yes | Yes | No |
| 29 CV | No | Yes | Yes | Yes |
| 30 CV | No | Yes | Yes | Yes |
| 31 CV | Yes | Yes | Yes | Yes |
| 32 CV | Yes | Yes | Yes | Yes |
| 33 CV | Yes | Yes | No | Yes |
| 34 CV | No | Yes | Yes | Yes |
| 35 CV | No | Yes | Yes | Yes |
| 36 CV | Yes | Yes | Yes | Yes |
| 37 CV | Yes | Yes | Yes | No |
| 38 CV | No | No | Yes | No |
| 39 CV | Yes | Yes | Yes | Yes |
| 40 CV | No | Yes | Yes | Yes |
| 41 CV | Yes | Yes | Yes | Yes |
| 42 CV | Yes | Yes | Yes | No |
| 43 CV | Yes | Yes | Yes | Yes |
| 44 CV | No | No | Yes | No |
| 45 CV | Yes | Yes | Yes | Yes |
| 46 CV | Yes | Yes | Yes | Yes |
| 47 CV | No | Yes | Yes | Yes |
| 48 CV | No | Yes | Yes | Yes |
| 49 CV | No | Yes | Yes | Yes |
| 50 CV | No | Yes | Yes | Yes |
| 51 CV | No | Yes | Yes | Yes |
| 52 CV | Yes | Yes | Yes | Yes |
| 53 CV | Yes | Yes | Yes | Yes |
| 54 CV | Yes | Yes | Yes | Yes |
| 55 CV | Yes | Yes | Yes | Yes |
| 56 CV | Yes | Yes | Yes | Yes |
| 57 CV | No | Yes | Yes | Yes |
| 58 CV | No | No | Yes | No |
| 59 CV | Yes | Yes | Yes | Yes |
| 60 CV | No | Yes | Yes | Yes |
| 61 CV | No | Yes | Yes | Yes |
| 62 CV | No | No | Yes | No |
| 63 CV | No | No | Yes | No |
| 64 CV | No | Yes | Yes | Yes |
| 65 CV | No | Yes | Yes | Yes |
| 66 CV | Yes | Yes | Yes | Yes |
| 67 CV | Yes | Yes | Yes | No |
| 68 CV | No | Yes | Yes | Yes |
| 69 CV | Yes | Yes | Yes | Yes |
| 70 CV | No | No | Yes | No |
| 71 CV | Yes | Yes | Yes | No |
| 72 CV | Yes | Yes | Yes | No |
| 73 CV | Yes | Yes | Yes | Yes |
| 74 CV | No | No | Yes | No |
| 75 CV | No | No | Yes | No |
| 76 CV | Yes | Yes | Yes | No |
| 77 CV | Yes | No | Yes | No |
| 78 CV | Yes | Yes | Yes | Yes |
| 79 CV | No | No | Yes | No |
| 80 CV | No | Yes | Yes | Yes |
| 81 CV | Yes | Yes | Yes | Yes |
| 82 CV | No | Yes | Yes | No |
| 83 CV | Yes | No | Yes | No |
| 84 CV | Yes | No | Yes | Yes |
| 85 CV | No | Yes | Yes | Yes |
| 86 CV | No | No | Yes | No |
| 87 CV | No | No | Yes | No |
| 88 CV | No | No | Yes | Yes |
| 89 CV | No | Yes | Yes | Yes |
| 90 CV | No | No | Yes | No |
| 91 CV | Yes | Yes | Yes | Yes |
| 92 CV | Yes | No | Yes | No |
| 93 CV | Yes | Yes | Yes | Yes |
| 94 CV | No | Yes | No | Yes |
| 95 CV | No | Yes | Yes | Yes |
| 96 CV | Yes | No | Yes | No |
| 97 CV | Yes | Yes | Yes | Yes |
| 98 CV | Yes | Yes | Yes | No |
| 99 CV | No | No | Yes | No |
| 100 CV | Yes | Yes | Yes | Yes |
| 101 CV | No | Yes | Yes | Yes |
| 102 CV | Yes | No | Yes | No |
| 103 CV | No | Yes | Yes | Yes |
| 104 CV | No | No | Yes | No |
| 105 CV | No | Yes | Yes | Yes |
| 106 CV | No | Yes | Yes | Yes |
| 107 CV | No | Yes | Yes | Yes |
| 108 CV | No | Yes | Yes | Yes |
| 109 CV | No | Yes | Yes | Yes |
| 110 CV | No | Yes | Yes | No |
| 111 CV | Yes | No | Yes | No |
| 112 CV | Yes | Yes | Yes | Yes |
| 113 CV | No | Yes | Yes | Yes |
| 114 CV | No | No | Yes | No |
| 115 CV | No | Yes | Yes | No |
| 116 CV | Yes | Yes | Yes | No |
| 117 CV | No | Yes | Yes | Yes |
| 118 CV | Yes | Yes | Yes | No |
| 119 CV | Yes | No | Yes | No |
| 120 CV | Yes | No | Yes | Yes |
| 121 CV | No | Yes | No | Yes |
| 122 CV | Yes | Yes | Yes | No |
| 123 CV | No | Yes | Yes | No |
| 124 CV | No | Yes | Yes | Yes |
| 125 CV | Yes | Yes | Yes | Yes |
| 126 CV | Yes | Yes | Yes | No |
| 127 CV | Yes | Yes | Yes | Yes |
| 128 CV | Yes | Yes | Yes | Yes |
| 129 CV | No | No | Yes | No |
| 130 CV | No | Yes | Yes | Yes |
| 131 CV | No | Yes | Yes | No |
| 132 CV | No | No | Yes | No |
| 133 CV | Yes | No | Yes | No |
| 134 CV | No | Yes | Yes | Yes |
| 135 CV | Yes | Yes | Yes | Yes |
| 136 CV | Yes | No | Yes | Yes |
| 137 CV | Yes | Yes | Yes | Yes |
| 138 CV | Yes | Yes | Yes | No |
| 139 CV | No | Yes | Yes | Yes |
| 140 CV | No | Yes | Yes | Yes |
| 141 CV | Yes | Yes | Yes | Yes |
| 142 CV | No | No | Yes | No |
| 143 CV | No | No | Yes | No |
| 144 CV | No | Yes | Yes | No |
| 145 CV | No | Yes | Yes | Yes |
| 146 CV | No | Yes | Yes | No |
| 147 CV | No | Yes | Yes | Yes |
| 148 CV | No | No | Yes | No |
| 149 CV | Yes | Yes | Yes | Yes |
| 150 CV | No | No | Yes | No |
| 151 CV | Yes | No | Yes | No |
| 152 CV | No | Yes | Yes | Yes |
| 153 CV | Yes | Yes | Yes | Yes |
| 154 CV | No | No | Yes | No |
| 155 CV | Yes | Yes | Yes | Yes |
| 156 CV | No | Yes | Yes | No |
| 157 CV | No | Yes | Yes | Yes |
| 158 CV | Yes | Yes | Yes | Yes |
| 159 CV | No | Yes | Yes | Yes |
| 160 CV | No | No | Yes | No |
| 161 CV | No | No | Yes | No |
| 162 CV | Yes | Yes | Yes | Yes |
| 163 CV | Yes | Yes | Yes | Yes |
| 164 CV | No | Yes | Yes | Yes |
| 165 CV | Yes | Yes | Yes | Yes |
| 166 CV | No | Yes | Yes | No |
| 167 CV | No | Yes | Yes | No |
| 168 CV | No | Yes | Yes | Yes |
| 169 CV | Yes | Yes | Yes | No |
| 170 CV | Yes | Yes | Yes | Yes |
| 171 CV | Yes | Yes | Yes | No |
| 172 CV | No | Yes | Yes | Yes |
| 173 CV | Yes | Yes | Yes | Yes |
| 174 CV | Yes | Yes | Yes | Yes |
| 175 CV | No | Yes | Yes | Yes |
| 176 CV | No | Yes | Yes | Yes |
| 177 CV | No | Yes | Yes | No |
