# Supplementary material for: Evaluation of hematological changes and immune response biomarkers as a prognostic factor in critical patients with COVID-19
Source: PLoS One. 2024 Feb 29;19(2):e0297490. doi: 10.1371/journal.pone.0297490 (PMC10903867; doi:10.1371/journal.pone.0297490)
Supplement: S5 Table — (DOCX) [file pone.0297490.s005.docx]

**Supporting information**

S5 Table. Inflammatory parameters and immune cell subsets ratios in COVID-19 ICU patients.

| **Sample** | **LMR** | **PLR** | **NLR** | **CLR** |
| --- | --- | --- | --- | --- |
| 1 CV | 3.10 | 422.46 | 13.26 | 0.31 |
| 2 CV | 1.71 | 706.06 | 23.96 | 0.16 |
| 3 CV | 1.03 | 637.46 | 25.91 | 0.32 |
| 4 CV | 2.21 | 42.66 | 9.40 | 0.14 |
| 5 CV | 2.02 | 218.74 | 47.72 | 0.04 |
| 6 CV | 3.29 | 165.36 | 6.81 | 0.02 |
| 7 CV | 0.47 | 766.55 | 92.69 | 0.01 |
| 8 CV | 5.01 | 210.33 | 8.69 | 0.11 |
| 9 CV | 3.87 | 401.57 | 4.69 | 0.01 |
| 10 CV | 3.86 | 165.73 | 2.41 | 0.00 |
| 11 CV | 2.70 | 679.19 | 10.93 | 0.12 |
| 12 CV | 1.29 | 596.34 | 12.20 | 0.19 |
| 13 CV | 1.76 | 144.37 | 10.11 | 0.10 |
| 14 CV | 2.16 | 258.88 | 23.17 | 0.13 |
| 15 CV | 2.85 | 96.22 | 9.86 | 0.04 |
| 16 CV | 1.00 | 575.12 | 30.93 | 0.04 |
| 17 CV | 5.01 | 348.11 | 8.69 | 0.18 |
| 18 CV | 1.44 | 268.49 | 11.64 | 0.18 |
| 19 CV | 1.56 | 256.89 | 14.33 | 0.17 |
| 20 CV | 1.01 | 758.54 | 47.09 | 0.78 |
| 21 CV | 1.99 | 298.28 | 26.75 | 0.05 |
| 22 CV | 1.33 | 320.71 | 14.77 | 0.06 |
| 23 CV | 0.60 | 683.15 | 29.97 | 0.13 |
| 24 CV | 2.25 | 72.76 | 12.38 | 0.10 |
| 25 CV | 3.26 | 136.93 | 17.09 | 0.21 |
| 26 CV | 1.40 | 154.76 | 12.42 | 0.11 |
| 27 CV | 0.90 | 704.32 | 33.11 | 0.17 |
| 28 CV | 3.57 | 98.35 | 2.71 | 0.00 |
| 29 CV | 2.17 | 274.11 | 18.30 | 0.12 |
| 30 CV | 1.45 | 640.98 | 42.35 | 0.42 |
| 31 CV | 1.37 | 252.68 | 4.73 | 0.02 |
| 32 CV | 0.84 | 461.36 | 51.14 | 0.34 |
| 33 CV | 1.73 | 166.63 | 14.54 | 0.00 |
| 34 CV | 1.19 | 226.09 | 12.80 | 0.14 |
| 35 CV | 1.56 | 536.95 | 11.50 | 0.16 |
| 36 CV | 1.17 | 363.62 | 20.60 | 0.20 |
| 37 CV | 3.56 | 208.87 | 10.16 | 0.14 |
| 38 CV | 1.26 | 607.09 | 18.86 | 0.05 |
| 39 CV | 0.68 | 238.35 | 9.02 | 0.01 |
| 40 CV | 5.26 | 159.84 | 3.01 | 0.00 |
| 41 CV | 1.42 | 230.11 | 24.54 | 0.27 |
| 42 CV | 1.40 | 455.03 | 23.30 | 0.03 |
| 43 CV | 2.03 | 1018.89 | 37.57 | 0.65 |
| 44 CV | 1.21 | 609.95 | 26.02 | 0.06 |
| 45 CV | 1.57 | 171.64 | 32.14 | 0.14 |
| 46 CV | 0.64 | 594.63 | 26.35 | 0.00 |
| 47 CV | 2.11 | 176.77 | 5.81 | 0.06 |
| 48 CV | 1.26 | 194.56 | 25.35 | 0.14 |
| 49 CV | 1.18 | 376.33 | 50.42 | 0.11 |
| 50 CV | 1.36 | 214.56 | 22.73 | 0.04 |
| 51 CV | 1.01 | 564.10 | 47.32 | 0.51 |
| 52 CV | 0.84 | 403.94 | 17.51 | 0.10 |
| 53 CV | 2.07 | 562.43 | 44.60 | 0.62 |
| 54 CV | 1.50 | 186.91 | 10.83 | 0.02 |
| 55 CV | 0.82 | 277.38 | 8.66 | 0.01 |
| 56 CV | 0.27 | 604.62 | 33.61 | 0.14 |
| 57 CV | 1.10 | 418.38 | 19.64 | 0.12 |
| 58 CV | 0.80 | 309.55 | 13.93 | 0.13 |
| 59 CV | 6.31 | 471.29 | 10.48 | 0.57 |
| 60 CV | 0.62 | 453.02 | 29.11 | 0.04 |
| 61 CV | 2.35 | 394.49 | 15.06 | 0.13 |
| 62 CV | 1.97 | 457.94 | 11.88 | 0.12 |
| 63 CV | 0.82 | 111.19 | 13.13 | 0.12 |
| 64 CV | 1.39 | 163.46 | 23.40 | 0.11 |
| 65 CV | 1.57 | 128.82 | 6.67 | 0.09 |
| 66 CV | 0.90 | 288.69 | 36.46 | 0.13 |
| 67 CV | 2.93 | 97.27 | 6.12 | 0.02 |
| 68 CV | 0.92 | 852.39 | 16.94 | 0.01 |
| 69 CV | 1.83 | 109.91 | 3.89 | 0.05 |
| 70 CV | 7.10 | 138.01 | 1.59 | 0.02 |
| 71 CV | 0.90 | 307.76 | 24.96 | 0.20 |
| 72 CV | 4.63 | 414.50 | 9.57 | 0.05 |
| 73 CV | 45.95 | 621.99 | 26.76 | 0.05 |
| 74 CV | 2.32 | 668.22 | 8.38 | 0.00 |
| 75 CV | 4.18 | 200.76 | 2.41 | 0.01 |
| 76 CV | 4.23 | 90.66 | 1.29 | 0.03 |
| 77 CV | 3.37 | 106.72 | 2.95 | 0.16 |
| 78 CV | 6.96 | 264.02 | 6.56 | 0.04 |
| 79 CV | 1.85 | 121.78 | 7.13 | 0.03 |
| 80 CV | 2.76 | 378.17 | 16.24 | 0.17 |
| 81 CV | 0.42 | 220.00 | 23.70 | 0.23 |
| 82 CV | 5.19 | 74.91 | 1.00 | 0.00 |
| 83 CV | 1.76 | 250.86 | 8.92 | 0.00 |
| 84 CV | 1.14 | 87.91 | 5.74 | 0.00 |
| 85 CV | 1.11 | 264.76 | 25.53 | 0.00 |
| 86 CV | 2.74 | 117.38 | 16.37 | 0.00 |
| 87 CV | 4.01 | 368.42 | 23.47 | 0.00 |
| 88 CV | 4.05 | 72.43 | 3.83 | 0.00 |
| 89 CV | 1.22 | 222.53 | 16.39 | 0.00 |
| 90 CV | 1.10 | 164.89 | 5.90 | 0.00 |
| 91 CV | 2.63 | 207.10 | 10.66 | 0.00 |
| 92 CV | 1.58 | 212.37 | 12.02 | 0.00 |
| 93 CV | 2.78 | 185.67 | 7.08 | 0.00 |
| 94 CV | 0.83 | 218.34 | 9.88 | 0.00 |
| 95 CV | 3.34 | 109.08 | 13.39 | 0.00 |
| 96 CV | 1.10 | 550.18 | 24.06 | 0.00 |
| 97 CV | 2.02 | 180.10 | 6.36 | 0.00 |
| 98 CV | 0.67 | 352.27 | 34.29 | 0.00 |
| 99 CV | 4.20 | 772.14 | 15.49 | 0.00 |
| 100 CV | 7.70 | 92.22 | 1.45 | 0.00 |
| 101 CV | 6.44 | 276.52 | 16.49 | 0.00 |
| 102 CV | 2.50 | 101.55 | 4.27 | 0.01 |
| 103 CV | 2.69 | 959.04 | 54.55 | 0.29 |
| 104 CV | 1.00 | 529.90 | 24.25 | 0.06 |
| 105 CV | 2.67 | 6.95 | 5.03 | 0.04 |
| 106 CV | 3.91 | 246.26 | 5.01 | 0.03 |
| 107 CV | 4.46 | 119.77 | 1.76 | 0.05 |
| 108 CV | 0.86 | 383.53 | 18.10 | 0.42 |
| 109 CV | 4.00 | 171.24 | 21.50 | 0.03 |
| 110 CV | 1.45 | 180.75 | 8.23 | 0.01 |
| 111 CV | 2.37 | 282.02 | 9.27 | 0.05 |
| 112 CV | 2.03 | 138.97 | 22.34 | 0.18 |
| 113 CV | 2.38 | 637.07 | 0.18 | 0.52 |
| 114 CV | 2.14 | 43.41 | 6.80 | 0.03 |
| 115 CV | 1.49 | 348.50 | 16.40 | 0.40 |
| 116 CV | 1.01 | 241.15 | 20.87 | 0.01 |
| 117 CV | 0.60 | 289.27 | 13.87 | 0.09 |
| 118 CV | 2.22 | 418.81 | 29.86 | 0.64 |
| 119 CV | 2.44 | 308.81 | 8.68 | 0.04 |
| 120 CV | 0.49 | 1685.63 | 73.51 | 0.61 |
| 121 CV | 1.42 | 152.87 | 22.73 | 0.17 |
| 122 CV | 0.72 | 45.46 | 15.05 | 0.14 |
| 123 CV | 1.09 | 127.54 | 10.54 | 0.01 |
| 124 CV | 1.09 | 365.26 | 16.53 | 0.26 |
| 125 CV | 0.93 | 212.96 | 11.91 | 0.24 |
| 126 CV | 1.73 | 194.16 | 14.27 | 0.00 |
| 127 CV | 1.40 | 451.75 | 33.45 | 0.16 |
| 128 CV | 1.64 | 707.25 | 32.26 | 0.18 |
| 129 CV | 1.07 | 1169.71 | 29.72 | 0.14 |
| 130 CV | 1.13 | 680.34 | 19.47 | 0.58 |
| 131 CV | 0.89 | 742.31 | 40.71 | 1.02 |
| 132 CV | 1.62 | 655.84 | 12.82 | 0.44 |
| 133 CV | 1.95 | 206.90 | 6.94 | 0.08 |
| 134 CV | 0.68 | 223.24 | 29.20 | 0.20 |
| 135 CV | 0.89 | 548.32 | 34.87 | 0.56 |
| 136 CV | 15.08 | 121.50 | 16.83 | 0.13 |
| 137 CV | 1.34 | 388.20 | 15.68 | 0.00 |
| 138 CV | 0.88 | 273.57 | 17.86 | 0.04 |
| 139 CV | 1.62 | 266.32 | 13.10 | 0.07 |
| 140 CV | 3.90 | 144.59 | 4.93 | 0.09 |
| 141 CV | 1.43 | 108.85 | 3.72 | 0.03 |
| 142 CV | 1.06 | 149.65 | 8.32 | 0.02 |
| 143 CV | 2.57 | 284.73 | 125.35 | 0.17 |
| 144 CV | 0.71 | 498.01 | 16.87 | 0.00 |
| 145 CV | 0.61 | 193.44 | 44.52 | 0.22 |
| 146 CV | 1.59 | 115.63 | 2.86 | 0.01 |
| 147 CV | 5.02 | 252.66 | 7.52 | 0.03 |
| 148 CV | 3.39 | 172.20 | 10.02 | 0.16 |
| 149 CV | 0.69 | 571.91 | 25.50 | 0.21 |
| 150 CV | 0.83 | 250.62 | 7.60 | 0.00 |
| 151 CV | 2.67 | 174.54 | 2.84 | 0.08 |
| 152 CV | 1.25 | 75.81 | 6.58 | 0.09 |
| 153 CV | 2.29 | 254.97 | 11.72 | 0.06 |
| 154 CV | 4.01 | 67.05 | 2.63 | 0.03 |
| 155 CV | 2.19 | 213.44 | 12.49 | 0.15 |
| 156 CV | 3.03 | 230.88 | 11.17 | 0.13 |
| 157 CV | 2.90 | 320.93 | 14.93 | 0.24 |
| 158 CV | 15.29 | 130.82 | 23.07 | 0.15 |
| 159 CV | 1.11 | 388.33 | 19.13 | 0.27 |
| 160 CV | 1.34 | 495.46 | 14.20 | 0.07 |
| 161 CV | 1.99 | 402.58 | 7.13 | 0.02 |
| 162 CV | 0.38 | 702.46 | 74.44 | 0.44 |
| 163 CV | 0.75 | 1158.49 | 75.69 | 0.60 |
| 164 CV | 1.45 | 290.20 | 21.33 | 0.20 |
| 165 CV | 1.86 | 695.05 | 7.64 | 0.21 |
| 166 CV | 2.48 | 189.62 | 5.67 | 0.19 |
| 167 CV | 0.86 | 346.57 | 15.19 | 0.06 |
| 168 CV | 1.15 | 308.63 | 19.07 | 0.29 |
| 169 CV | 2.26 | 38.30 | 4.81 | 0.04 |
| 170 CV | 0.60 | 238.13 | 10.82 | 0.06 |
| 171 CV | 1.96 | 556.42 | 16.97 | 0.09 |
| 172 CV | 1.50 | 139.62 | 109.43 | 0.04 |
| 173 CV | 1.87 | 667.71 | 69.30 | 0.15 |
| 174 CV | 5.87 | 76.05 | 7.58 | 0.04 |
| 175 CV | 0.22 | 656.15 | 44.74 | 0.21 |
| 176 CV | 6.07 | 431.47 | 13.58 | 0.64 |
| 177 CV | 4.15 | 360.92 | 9.41 | 0.23 |
